# The role of spinal injections towards optimizing patient selection for spinal surgery: A proof-of-concept study in 176 lower back pain patients

**DOI:** 10.1186/s13037-024-00414-y

**Published:** 2024-10-31

**Authors:** Anna Voelker, Katharina Kroboth, Christoph-Eckhard Heyde

**Affiliations:** Department for Orthopedics, Trauma and Plastic Surgery, University Hospital Leipzig AöR, Liebigstraße 20, 04103 Leipzig, Germany

**Keywords:** Degenerative lumbar spine disease, Spinal injections, Conservative spine treatment, Therapeutic spinal interventions

## Abstract

**Background:**

Managing degenerative lumbar diseases is challenging due to the complexity of identifying symptom causes, especially when multiple pathologies coexist. This study evaluated the impact of patient-specific lumbar spine infiltrations on therapeutic strategies in patients with multiple spinal pathologies (MSP) or specific spinal pathologies (SSP).

**Methods:**

A retrospective cohort of 176 patients treated for subacute or chronic lumbar pain with targeted spinal infiltrations was analyzed. Patients were categorized based on the presence of MSP or SSP. The primary endpoint was the relief of lumbar spine-specific symptoms following each infiltration. Secondary endpoints included epidemiological factors and comorbidities, while tertiary endpoints focused on post-treatment recommendations and performed treatments.

**Results:**

High rates of spinal pain (97.1% in both groups) and radiating symptoms (88.2% in SSP and 92.3% in MSP) were reported. Psychological conditions were significantly more prevalent in female patients (19.4% vs. 7.7%, *p* = 0.0307), whereas hip osteoarthritis was more common in male patients (20.5% vs. 9.2%, *p* = 0.0490). Among all infiltration types, lumbar transforaminal injections were the most effective, leading to pain reduction in 80.1% of SSP patients and 72.2% of MSP patients. Facet joint and sacroiliac joint infiltrations also frequently resulted in pain reduction in both groups. Overall, conservative treatment was recommended for most patients (73.3%), while only 22.7% of all evaluated patients were recommended for surgical intervention. Additionally, seven patients received a hip prosthesis.

**Conclusions:**

Patient-specific lumbar spine infiltrations effectively relieve pain, support therapeutic decision-making, and tend to favor conservative treatment approaches. These findings highlight the role of infiltration therapies in managing both mixed and specific lumbar spine pathologies, suggesting their potential to reduce the need for surgical interventions.

## Introduction

Degenerative spine disorders represent a major global health issue, affecting a significant portion of the adult population and contributing considerably to disability worldwide. As populations age, the prevalence of degenerative spine disorders is projected to rise, placing an increasing strain on healthcare systems [1]. These disorders include a range of conditions such as intervertebral disc degeneration, facet joint osteoarthritis, spinal stenosis, and degenerative spondylolisthesis [2]. Their etiology is multifactorial, involving genetic predisposition, age-related changes, mechanical stress, and lifestyle factors [3]. These conditions often result in chronic pain, reduced mobility, and diminished quality of life, requiring a comprehensive diagnostic and management approach that spans both conservative and surgical treatments.

However, selecting the optimal therapy for patients with degenerative spine disorders remains a significant challenge for clinicians. The complex interaction between symptoms, imaging results, and individual patient characteristics often complicates the decision of whether conservative management or surgical intervention is more appropriate.

Spinal symptoms can be caused by a specific pathology or by an accumulation of various pathologies, particularly as degenerative conditions become more common with increasing age. While younger patients often have isolated issues, the aging process frequently leads to a complex array of degenerative changes in the lumbar spine [4, 5]. These changes often coexist, resulting in multiple pathologies visible on diagnostic imaging, which can complicate the clinical picture [6].

The sacroiliac joint (SIJ) can also be a source of symptoms that resemble those of lumbar spine pathologies. Furthermore, it has been reported that up to 30% of patients with lumbar complaints also experience pain in the SIJ region [7]. When the SIJ is the source of pain, it can cause symptoms that radiate into the lower extremities, often leading to confusion with lumbar radiculopathies. These pseudo-radicular pain syndromes are not always distinguishable from true lumbar pathologies due to their similar clinical presentation. Therefore, it is crucial to consider the SIJ as a potential cause of back pain and to perform appropriate diagnostic measures to identify the exact source of pain and initiate adequate treatment [8].

Hip disorders can also present with symptoms that mimic sciatica, complicating the diagnosis. Conditions such as hip osteoarthritis or greater trochanter pain syndrome can cause referred pain that may be mistaken for radicular pain [9, 10]. This overlap of symptoms can lead to misdiagnosis, as patients with arthritic changes often also have degenerative changes in the lumbar spine visible on imaging, although the primary problem originates from the hip. It is therefore important to consider hip pathologies in the differential diagnosis of sciatica-like symptoms and to use appropriate clinical assessments and imaging techniques to ensure accurate diagnosis and effective treatment.

Another challenge in the diagnosis and treatment of lumbar spine disorders is the occurrence of chronic pain following surgery, which can affect 8–40% of cases. This pain is often due to persistent structural issues that were not resolved surgically, such as bony, discogenic, or ligamentous problems. Inadequate decompression of the nerve roots and spinal canal can also contribute to chronic pain after lumbar surgery [11, 12]. When the cause of pain remains unclear, diagnostic infiltrations before a planned surgery sharpens the indication and thus can help to prevent the development of chronic postoperative pain. By identifying the pain-inducing structures preoperatively, targeted surgical treatments can be implemented, potentially avoiding chronic postoperative complications [13–15].

Infiltration therapies for the lumbar spine can bridge the gap between conservative treatment and surgical intervention. Specific and nonspecific infiltrations in this area must be differentiated. Specific infiltrations target precise anatomical structures, such as nerve roots or facet joints, and are used both diagnostically and therapeutically. Nonspecific infiltrations, in contrast, aim to reduce general inflammation and pain in a broader area. Although the primary application of spinal infiltrations is therapeutic, targeted infiltrations are also used to diagnose the underlying pathology causing the symptoms [16]. Pain relief through diagnostic infiltration therapies can improve patient functionality and reduce pain, thereby enabling a more targeted application of active treatments within the conservative treatment framework [17]. In some cases, this can help avoid surgical interventions [18]. Additionally, the analysis of the patient’s response to targeted infiltrations can support the planning of surgical interventions in the lumbar spine [19].

The aim of this study was to assess the diagnostic and therapeutic utility of patient-specific infiltrations of the lumbar spine, sacroiliac joint, and/or hip joint in patients with spinal disorders treated at a specialized spine center, and to evaluate their impact on subsequent therapeutic decision-making, particularly in choosing between conservative and surgical approaches.

## Materials and methods

### Ethics statements

This study was approved by the local ethics committee (reference number: 459/17-ek) and adhered to the ethical standards of the Declaration of Helsinki. The consent for retrospective data analysis was granted by the patients through the hospital admission contract.

### Study design and population

This retrospective single-center study was conducted at a large, specialized university hospital spine center in Germany over a one-year period in 2015. The study analyzed data from consecutive patients with subacute (6–12 weeks) and chronic (> 12 weeks) back pain who had documented pathology in lumbar spine imaging.

**Fig. 1:**
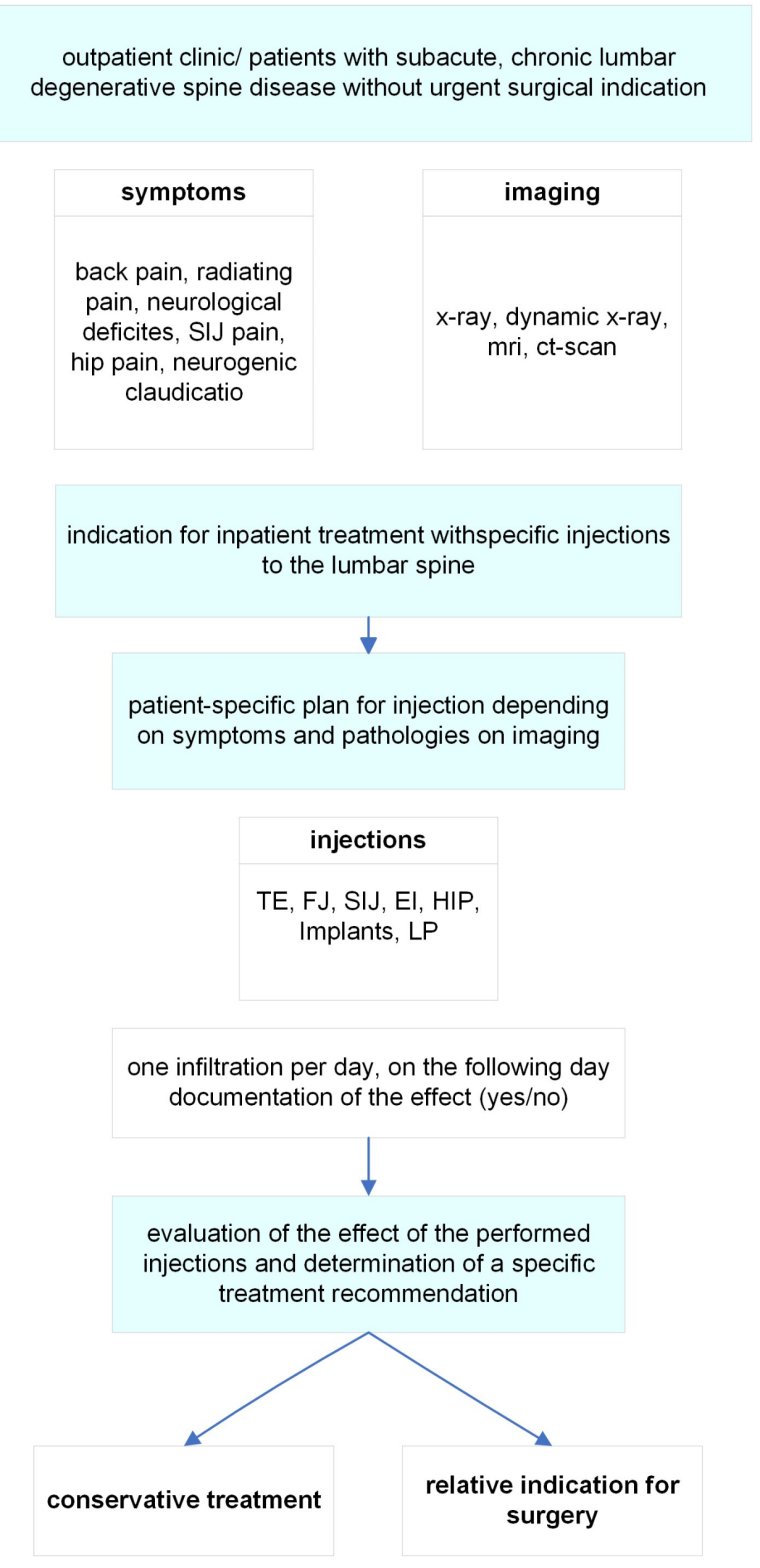
Therapeutic algorithm for inpatient conservative spine therapie

A tailored inpatient treatment protocol was implemented, involving a stepwise approach where only one specific type of injection was administered each day based on the patient’s symptoms and imaging findings. If patients exhibited additional abnormalities in the sacroiliac joint (SIJ) or hip joint during clinical or imaging assessments, an injection was also performed in these areas. The complete in-hospital treatment algorithm, outlining the decision-making process and injection schedule, is detailed in Fig. 1. Surgical treatment was recommended when there was a concordance between clinical symptoms, imaging findings, and response to infiltrations, indicating a specific pathology amenable to surgical intervention, and when previous conservative measures had not provided adequate relief. Following inpatient injection treatment, patients are regularly followed up in the spine clinic every 3 months.

Exclusion criteria included patients with acute immobilizing pain or acute neurological deficits requiring immediate hospitalization, as well as those with fractures, tumors, or infections, and those who were candidates for multimodal therapy based on an assessment of factors such as medication dependency, psychological comorbidities, and the need for comprehensive pain management.

### Analyzed parameters

Patients were stratified into cohorts based on their diagnosis. One group included those with multiple spinal pathologies (MSP), referred to as mixed pathologies, which often occur together, such as facet joint hypertrophy, spinal canal stenosis, degenerative spondylolisthesis, and changes in the sacroiliac joint and hip joint. The rationale for this grouping is that in cases of mixed pathologies, it is often unclear which pathology is the most symptomatic structure causing the patient’s pain. The other group consisted of patients with specific spinal pathologies (SSP), where a solitary identifiable pathology, such as a herniated disc, lumbar spinal stenosis, or lumbosacral spondylolisthesis, was present within the lumbar spine. In these cases, the clinical symptoms typically align well with the imaging findings, making it easier to identify the underlying cause of the pain.

These patients were admitted to the hospital to initiate an extended conservative therapy. Infiltration treatments were performed according to a therapy plan individually tailored to the patient’s specific symptoms and pathologies. Only one type of injection was administered per day, which included procedures such as facet joint (FJ) injections (limited to one segment per day), epidural interlaminar (EI) injections, transforaminal epidural injections (TEI), lumbar paraspinal (LP) injection, as well as sacroiliac joint (SIJ) and hip joint (HIP) infiltrations.

All infiltrations were performed using specific techniques and image guidance to ensure precise needle placement and medication delivery. TEI were conducted under CT guidance, utilizing a combination of local anesthetic and glucocorticoid. FJ and SIJ were both performed under X-ray guidance, with FJ injections using only local anesthetic, while SIJ injections employed a combination of local anesthetic and glucocorticoid. EI and LP were administered freehand, without image guidance, both using a mixture of local anesthetic and glucocorticoid. HIP were carried out under X-ray guidance, also using a combination of local anesthetic and glucocorticoid.

The primary endpoints of this study were twofold: firstly, to identify the presence of specific lumbar spine-related symptoms, and secondly, to assess the effectiveness of injections in reducing pain across both patient groups (MSP and SSP). Pain relief was evaluated using a simple, binary patient-reported outcome measure. On the morning following each injection, patients were asked to indicate whether the procedure had led to a reduction in their symptoms (yes/no).

Secondary outcomes included the evaluation of epidemiological factors and comorbidities in patients with MSP and SSP. Additionally, data on demographics, length of hospital stay, history of previous lumbar back surgery, and selected comorbidities—both general and those potentially related to low back pain—were collected and analyzed.

The tertiary endpoints of the study focused on post-treatment recommendations and the treatments actually performed. The evaluation of the treatments performed was conducted at least one year after the initial therapeutic recommendations and analyzed in terms of conservative or surgical interventions in the lumbar spine.

### Statistical analysis

Statistical analyses were conducted using Prism software, version 10 (GraphPad Software, La Jolla, CA, USA). Data are presented as mean ± standard deviation. The Shapiro-Wilk test was used to assess the normality of the data. For normally distributed data, the Student’s t-test was applied. For non-normally distributed data, such as age, the Mann-Whitney U test was employed. Differences in categorical variables, including comparisons between sexes and between the MSP and SSP groups, were assessed using Fisher’s exact test. Statistical significance was set at *p* < 0.05.

## Results

In total, 176 patients who underwent individualized conservative management for spinal pain at a single-level spine facility were analyzed. The cohort included 78 men and 98 women, with a mean age of 64 years (range 23–88). Patients were categorized into two groups: 142 with MSP and 34 with SSP. Further descriptive data, including length of hospital stay and pre-operated status, are detailed in Table 1.

**Table 1:** Descriptive patient data

	All patients (*n* = 176)	Men (*n* = 78)	Women (*n* = 98)	*p*-value	MSP (*n* = 142)	SSP (*n* = 34)	*p*-value
Age (yrs; mean, range)	64 (23–87)	64(30–87)	64 (23–88)	0.9425	65 (29–88)	60 (23–87)	0.1041
Length of hospital stay (days)	4.591 (1–14)	4.9 (1–14)	4.3 (1–11)	0.1837	4.5 (1–14)	5.2 (2–14)	0.0937
Previous spine surgery	68	26 (38.2%)	42 (61.8%)	0.2063	57 (83.8%)	11 (16.2%)	< 0.0001*

Descriptive patient data comparing demographics and clinical characteristics between men and women, and between patients with multiple spinal pathologies (MSP) and specific spinal pathology (SSP). P-values indicate the statistical significance of differences between compared groups (men vs. women and MSP vs. SSP), with *p* < 0.05 considered statistically significant. MSP, multiple spinal pathologies; SSP, specific spinal pathology; Previous spine surgery, patients who had undergone spinal surgical procedures prior to their current treatment. *Indicates statistical significance (*p* < 0.05).

### Clinical symptoms

Clinical symptoms were observed in both MSP (*n* = 142) and SSP (*n* = 34) patients, the latter including subgroups of disc prolapse (*n* = 16), lumbar spinal stenosis (LSS, *n* = 14), and lumbosacral spondylolisthesis (*n* = 4). As detailed in Table 2, a high prevalence of back pain was noted across both groups, affecting 172 patients (97.7%), with 97.9% in the MSP group and 100% in the SSP group. Radiating pain was also common, occurring in 161 patients (91.5%), with similar prevalence between MSP (92.3%) and SSP (93.8%) groups.

Motor deficits were present in 36 patients (20.5%), with a slightly higher incidence in the disc prolapse subgroup (25.0%). Sensory deficits were reported in 64 patients (36.4%), with the highest occurrence in the disc prolapse subgroup (50.0%). A detailed sub analysis of the severity, cause, and duration of these chronic neurological symptoms was not performed as part of this study. SIJ pain was noted in 101 patients (57.4%) overall, with a particularly high prevalence in the lumbosacral spondylolisthesis subgroup (100%). Lastly, neurogenic claudication was observed in 62 patients (35.2%), occurring more frequently in the MSP group (39.4%) than in the SSP group (6.3% in disc prolapse and 35.7% in lumbar spinal stenosis) (Table 2).

### Type of spinal injections

The analysis of interventional treatments revealed diverse spinal injection therapies, as detailed in Table 2. Facet joint (FJ) and SIJ injections were the most common therapies, administered in 118 (67.0%) and 117 (66.5%) of all patients, respectively. Transforaminal epidural injections (TEI) were administered in 93 cases (52.8%), with the highest usage in the disc prolapse subgroup (87.5%). Epidural interlaminar (EI) injections were performed in 49 cases (27.8%), while lumbar paraspinal (LP) injections, implants, and hip joint (HIP) injections were the least commonly administered therapies (Table 2).

**Table 2:** Clinical symptoms and type of spinal injections

Clinical symptoms	All patients (*n* = 176)	MSP (*n* = 142)	SSP (*n* = 34)		
			Disc prolapse (*n* = 16)	Lumbar spinal stenosis (*n* = 14)	Lumbosacral spondylolisthesis (*n* = 4)
Back pain	172 (97.7%)	139 (97.9%)	16 (100%)	13 (92.9%)	4 (100%)
Radiating pain	161 (91.5%)	131 (92.3%)	15 (93.8%)	11 (78.6%)	4 (100%)
Motor deficite	36 (20.5%)	30 (21.1%)	4 (25.0%)	1 (7.1%)	1 (25.0%)
Sensory deficite	64 (36.4%)	50 (35.2%)	8 (50.0%)	5 (35.7%)	1 (25.0%)
SIJ pain	101 (57.4%)	85 (59.9%)	4 (25.0%)	8 (57.1%)	4 (100%)
Hip pain	32 (18.2%)	27 (19.0%)	2 (12.5%)	3 (21.4%)	-
Neurogenic claudicatio	62 (35.2%)	56 (39.4%)	1 (6.3%)	5 (35.7%)	-
**Injection type**					
TEI	93 (52.8%)	72 (50.7%)	14 (87.5%)	4 (28.6%)	3 (75.0%)
FJ	118 (67.0%)	100 (70.4%)	6 (37.5%)	9 (64.3%)	3 (75.0%)
SIJ	117 (66.5%)	99 (69.7%)	5 (31.3%)	9 (64.3%)	4 (100%)
EI	49 (27.8%)	43 (30.3%)	-	5 (35.7%)	1 (25.0%)
LP	5 (2.8%)	3 (2.1%)	-	2 (14.3%)	-
Implants	10 (5.7%)	7 (4.9%)	-	3 (21.4%)	-
HIP	8 (4.5%)	6 (4.2%)	-	2 (14.3%)	-

MSP, multiple spinal pathologies; SSP, specific spinal pathology; TEI, transforaminal epidural injection; FJ, facet joint; SIJ, sacroiliac joint; EI, epidural interlaminar; HIP, hip joint; Implants, injection of pre-existing spinal hardware such as screws; LP, lumbar paraspinal

### Comorbidities

Table 3 outlines the comorbidity prevalence in the study cohort. Arterial hypertension was the most prevalent general comorbidity, affecting 65.3% of patients, with no significant sex difference (*p* = 0.7528). Diabetes mellitus was present in 18.2% of patients, being more common in men than in women, though not significantly (*p* = 0.0763). Hypercholesterolemia and polyneuropathy showed similar non-significant sex differences. Psychological diseases were significantly more common in women (19.4% vs. 7.7%; *p* = 0.0307). Smoking data were incomplete, but among 155 patients, 35.5% were smokers, with no significant sex difference (*p* = 0.3142). Hip osteoarthritis prevalence was significantly higher in men (20.5% vs. 9.2%; *p* = 0.0490), while no significant differences were observed in rheumatic diseases, osteoporosis, or average BMI between sexes or between the MSP and SSP groups. Additionally, no significant differences in general comorbidity prevalence were found between the MSP and SSP groups (Table 3).

**Table 3:** General comorbidities and comorbidities with possible influence on lumbar spine symptoms

Comobidities	All patients (*n* = 176)	Men (*n* = 78)	Women (*n* = 98)	*p*-value	MSP (*n* = 142)	SSP (*n* = 34)	*p*-value
**General comobidities**							
Arterial hypertension	115 (65.3%)	52 (66.7%)	63 (64.3%)	0.7528	97 (68.3%)	18 (52.9%)	0.1094
Diabetes mellitus	32 (18.2%)	19 (24.4%)	13 (13.3%)	0.0763	29 (20.4%)	3 (8.8%)	0.1414
Hypercholesterolemia	41 (32.3%)	18 (23.1%)	23 (23.5%)	> 0,9999	34 (23.9%)	7 (20.6%)	0.8225
Polyneuropathy	11 (6.3%)	6 (7.7%)	5 (5.1%)	0.541	11 (7.7%)	0	0.126
Psychological disease	25 (14.2%)	6 (7.7%)	19 (19.4%)	0.0307*	21 (14.8%)	4 (11.8%)	0.7889
Smoking* (*n* = 155)	55 (19.5%)*	21/68 (30.9%)²	34/87 (39.1%)²	0.3142	42 (124)*	13 (31)*	0.409
**Comorbidities affecting the spine**							
Rheumatic diseases	11 (6.3%)	4(5.1%)	7 (7.1%)	0.7569	8 (5.6%)	3 (8.8%)	0.4464
Hip osteoarthritis	25 (14.2%)	16 (20.5%)	9 (9.2%)	0.0490*	23 (16.2%)	2 (5.8%)	0.1719
Osteoporosis	16 (9.1%)	5 (6.4%)	11 (11.2%)	0.3038	12 (8.5%)	4 (11.8%)	0.5163
Obesity (mean BMI kg/m^2^, range)	28.35 (20–51)	29 (21–51)	27,84 (20–39)	0.136	28.37 (20–51)	28.2 (21–38)	0.8745

MSP, multiple spinal pathologies; SSP, specific spinal pathology; BMI, body mass index.

² For comorbid smoking, data were collected in only 155 patients.

### Effectiveness of injection therapy on pain reduction

The effect of infiltration therapies on pain reduction is shown in Table 4. In the MSP group (*n* = 142), transforaminal epidural injections (TEI) were administered in 72 cases, with a notable pain reduction in 72.2% of cases. Facet joint (FJ) and SIJ injections were the most common treatments, administered in 100 (61.0%) and 99 (45.5%) cases, respectively. Epidural interlaminar (EI) injections were less common, administered in 43 cases with a pain reduction rate of 53.5%. Lumbar paraspinal (LP) injections and implants were used sparingly, administered in 3 and 7 cases, with pain reduction rates of 33.3% and 57.1%, respectively. Hip joint (HIP) injections, the least frequent treatment, were administered in 6 cases, with a pain reduction rate of 66.7%.

In the SSP group (*n* = 34), the overall response to TEI was higher, with a pain reduction rate of 80.1% across 21 cases. Notably, all 3 cases of TEI in the lumbosacral spondylolisthesis subgroup resulted in pain relief. FJ and SIJ injections were less effective in the SSP group than in the MSP group, with pain reduction rates of 42.9% each. The response to EI injections was also lower in the SSP group, with a pain reduction rate of 33.3% among 6 cases. The disc prolapse subgroup had the highest response rate to TEI at 85.7%. For FJ injections, the lumbar spinal stenosis subgroup showed a higher response rate (66.7%) compared to the disc prolapse subgroup (33.3%). SIJ injections were most effective in the lumbar spinal stenosis subgroup, with a pain reduction rate of 55.6%. LP injections and implants were rarely used but showed pain reduction rates of 50.0% in the SSP group (Table 4).

**Table 4:** Effects of injections in terms of pain relief

		MSP	SSP			
Injection type	Number of injections/ successful injections^†^	Overall (*n* = 142)	Overall (*n* = 34)	Disc prolapse (*n* = 16)	Lumbar spinal stenosis (*n* = 14)	Lumbosacral spondylolisthesis (*n* = 4)
**TEI**	Total n	72	21	14	4	3
	Sucessful n (%)	52 (72.2%)	17 (80.1%)	12 (85.7%)	2 (50.0%)	3 (100%)
**FJ**	Total n	100	18	6	9	3
	Sucessful n (%)	61 (61.0%)	9 (42.9%)	2 (33.3%)	6 (66.7%)	1 (33.3%)
**SIJ**	Total n	99	18	5	9	4
	Sucessful n (%)	45 (45.5%)	9 (42.9%)	2 (40.0%)	5 (55.6%)	2 (50.0%)
**EI**	Total n	43	6	-	5	1
	Sucessful n (%)	23 (53.5%)	2 (33.3%)	-	2 (40.0%)	-
**LP**	Total n	3	2	-	2	-
	Sucessful n (%)	1 (33.3%)	1(50.0%)	-	1 (50.0%)	-
**Implants**	Total n	7	3	-	3	-
	Sucessful n (%)	4 (57.1%)	1(33.3%)	-	1 (33.3%)	-
**HIP**	Total n	6	2	-	2	-
	Sucessful n (%)	4 (66.7%)	-	-	-	-

^†^Successful injection determined by the patient’s endorsement of a reduction in pain (yes/ no)

MSP, multiple spinal pathologies; SSP, specific spinal pathology; TE, transforaminal epidural; FJ, facet joint; SIJ, sacroiliac joint; EI, epidural interlaminar; Hip, hip joint; Implants, injection of spinal implants; LP, lumbar paraspinal

### Recommended and performed treatment after evaluation patient specific injections

Across all patients, 73.3% (*n* = 129) were recommended for conservative treatment, while 26.7% (*n* = 47) were advised to undergo surgery, with a significant difference (*p* < 0.0001). Similarly, 77.3% (*n* = 136) of patients actually received conservative treatment, and 22.7% (*n* = 40) underwent surgery, also with a significant difference (*p* < 0.0001). Among patients with multiple spinal pathologies (MSP), 78.9% (*n* = 112) were recommended for conservative treatment, and 21.1% (*n* = 30) for surgery, with 79.6% (*n* = 113) and 20.4% (*n* = 29), respectively, following through with these treatments (*p* < 0.0001 for both). No significant difference was found between recommended and performed surgical treatments (*p* > 0.9999). For specific spinal pathologies (SSP), recommendations were evenly split (50% for both treatments), but 79.4% (*n* = 27) received conservative treatment, and 20.5% (*n* = 7) underwent surgery (*p* = 0.0053). No significant difference was observed between recommended and performed surgical treatments in this group (*p* = 0.0969) (Table 5).

**Table 5:** Therapeutic recommendations and performed treatment after patient-specific inpatient injection therapy

	Recommendation			Performed			Surgery Rec./Perf.
Patients	Conservative treatment	Relative indication for surgery	p-value	Conservative treatment	Surgery	p-value	*p*-value
All patients (*n* = 176)	129 (73.3%)	47 (26.7%)	< 0.0001	136 (77.3%)	40 (22.7%)	< 0.0001	0.55
MSP (*n* = 142)	112 (78.9%)	30 (21.1%)	< 0.0001	113 (79.6%)	29 (20.4%)	< 0.0001	> 0.9999
SSP (*n* = 34)	17 (50.0%)	17 (50.0%)	> 0.9999	27 (79.4%)	7 (20.5%)	0.0053	0.0969

*p < 0.05

MSP, multiple spinal pathologies; SSP, specific spinal pathology; Surgery Rec/ Perf., Surgery Recommendation Performed

## Discussion

Back pain and radicular pain are the primary symptoms of degenerative spinal diseases [20, 21]. This aligns with our findings, which showed a high prevalence of these symptoms in both the MSP and SSP groups. These primary symptoms occurred with similar frequency in both groups, highlighting the widespread nature of pain in these conditions and suggesting that these symptoms are not specific to a single pathology. According to our analysis, patients from various groups reported similar symptoms, further emphasizing the partially nonspecific nature of back and radicular pain in the context of diagnosis. However, our data clearly show that specific pathologies with potential nerve root compression more frequently lead to radicular pain and sensory deficits, as seen in the disc prolapse group.

Although no significant differences were found in general or spinal-related comorbidities between men and women or between the MSP and SSP groups, a notable difference was observed in the prevalence of psychological conditions. Women in our patient cohort had a significantly higher prevalence of psychological disorders compared to men, highlighting the importance of gender-specific treatment strategies. The interaction between chronic pain and psychological disorders, particularly in women, is well documented, with these conditions frequently co-occurring [22, 23]. This underscores the influence of sex on pain perception, reporting, and the development of psychological conditions, suggesting that women may experience a more complex interplay between physical and psychological factors, necessitating an integrated, gender-sensitive approach to treatment.

Infiltrations in the lumbar spine are essential for both diagnostics and therapy. Recent reviews have demonstrated that transforaminal epidural injections (TEI), as well as facet joint (FJ) and sacroiliac joint (SIJ) infiltrations, can effectively reduce pain. TEI, in particular, has proven valuable in treating radicular pain caused by nerve compression [18]. Our findings support this, as most patient groups in our study, except for the lumbar spinal stenosis subgroup, reported pain relief following TEI. This response suggests that the symptoms are primarily caused by nerve compression at the specifically targeted nerve root. The positive response to TEI also serves as a diagnostic tool, confirming the involvement of the infiltrated nerve root in the pain generation and thereby guiding subsequent therapeutic decisions.

However, the accuracy and effectiveness of TEI are highly dependent on the precision of the injection technique [24] identified several factors that can lead to false-negative results, including inadequate infiltration and intrapleural injections. Additionally, they noted that false-positive outcomes can occur when excessive injectate volume causes unintended spread into the epidural space or adjacent symptomatic levels. To ensure accurate needle placement and minimize these risks, TEI should be performed under CT guidance. These findings underscore the critical importance of precise technique, careful control of injection volume, and the use of imaging guidance to ensure both diagnostic accuracy and therapeutic efficacy with TEI.

Furthermore [16], conducted a systematic review and found limited evidence supporting the accuracy of selective nerve root injections, with a 50% pain reduction being the reference standard in the evaluated studies. In cases where symptoms are unclear and do not correlate well with imaging findings, the authors recommend using TEI as a possible diagnostic tool to help clarify the source of pain.

The evidence for the effectiveness of caudal, interlaminar, and transforaminal epidural injections (TEI) in the treatment of radicular pain, particularly concerning long-term improvements, is classified as Level II, with no significant differences observed among these approaches [17, 25]. The use of interlaminar epidural injections, particularly in the MSP group and LSS subgroup, demonstrated a significant positive effect on pain reduction. In our patient cohort, a glucocorticoid was always used in combination with a local anesthetic for EI, except in cases where contraindications were present. According to the literature, this combination enhances the effectiveness of the injections compared to the use of a local anesthetic alone, highlighting the synergistic benefit of this approach, which aligns with current best practices [26].

In the current literature, there is conflicting evidence regarding the diagnostic accuracy of facet joint (FJ) infiltrations. However, there is strong evidence supporting the use of image-guided FJ infiltrations. Boswell et al. demonstrated that a 75% reduction in pain can be achieved with these infiltrations, though they reported a false-positive rate of 25–44% [27]. In our study, patients from the MSP and LSS groups, specifically 66.7% and 61%, reported a positive effect on pain relief following FJ infiltration, suggesting that the facet joints are likely the source of symptoms in these patient groups. In our practice, FJ injections are performed under fluoroscopic guidance, with only one segment injected per day to ensure precision and enhance diagnostic validity.

Complaints related to the structures of the sacroiliac joint (SIJ) were routinely observed in both the MSP and SSP groups. Therefore, patients received infiltrations targeting the dorsal structures of the sacroiliac joint, a critical area often involved in lower back pain. A reduction in symptoms was observed in both groups following these infiltrations, indicating the effectiveness of this approach in treating SIJ-related pain. The occurrence of pain in this region can be anatomically explained by a study conducted by Steinke et al., which showed that branches of the dorsal rami of L5 and S1 pass through the inner sacral ligaments, while the outer sacral ligaments are traversed by branches from S1 to S4 [28]. This complex nerve supply may contribute to the intricate pain patterns observed in patients with SIJ pain. Furthermore, a systematic review by Simopoulos et al. provided compelling evidence for the diagnosis of SIJ pain through the use of diagnostic blocks in this region, highlighting the importance of targeted infiltrations for accurate diagnosis and effective treatment [8]. Given these anatomical and clinical insights, the role of SIJ infiltrations as both a diagnostic and therapeutic tool is emphasized in clinical practice, underscoring their value in the comprehensive management of patients with lower back pain.

Notably, our study found a high success rate of SIJ infiltrations even in the SSP group, which might initially seem counterintuitive. However, this aligns with established evidence showing a significant overlap between lumbar spine pathologies and SIJ symptoms. Research has consistently shown that a substantial proportion of patients with lumbar spine disorders also experience SIJ-related symptoms, which can often be the primary source of their pain. Sembrano and Polly (2009) reported that in 26.6% of patients presenting with low back pain, the SIJ was the primary pain source. This high comorbidity rate explains the observed effectiveness of SIJ infiltrations in SSP patients. Additionally, Slipman et al. demonstrated that SIJ pain can radiate to areas commonly associated with lumbar spine disorders, further complicating the clinical picture [29]. Cohen et al. highlighted in a comprehensive review that the overlap between SIJ pain and lumbar pathologies is a common clinical challenge [30]. These findings emphasize the importance of considering SIJ involvement even in patients with apparent specific spinal pathologies, supporting our approach of incorporating SIJ infiltrations into the diagnostic and therapeutic algorithm for both MSP and SSP patients.

Seven out of 32 patients with pseudo-radicular lumbar pain and hip pain who responded positively to hip joint (HIP) infiltration and hat radiological signs of hip osteoarthritis were recommended for total hip arthroplasty, despite the presence of additional degenerative changes in the lumbar spine. This finding aligns with current literature. Clarius et al. recommended prioritizing hip surgery, citing an increased risk of HIP dislocation if spinal surgery is performed first [31]. Similarly, Ran et al. demonstrated that patients with degenerative changes in both the hip and lumbar spine experienced improvements in back pain following total hip arthroplasty [32, 33]. These results support the notion that hip pathology can contribute to lumbar symptoms, and that addressing hip issues may have a positive impact on lumbar spine-related pain.

Our findings and approach are further supported by the growing recognition of the “hip-spine syndrome” or “hip-spine connection” in recent years [34, 35]. The hip-spine syndrome explains why some of our patients with apparent lumbar pathologies responded well to hip infiltrations and were ultimately recommended for hip surgery. It also highlights the necessity of our comprehensive diagnostic approach, which includes both spinal and hip evaluations and interventions. By addressing both the lumbar spine and hip through our infiltration protocol in symptomatic patients, we were able to more accurately identify the primary pain generator and recommend the most appropriate treatment, even in cases where initial presentation suggested a primarily spinal origin of symptoms.

The current study results underscore the urgent need for personalized treatment regimens in patients with low back pain and various degenerative spinal pathologies. This highlights the varying effectiveness of the spinal injections we administered across different patient groups. Spinal injections can accurately identify pain-inducing structures in various pathologies, enabling targeted interventions in cases requiring surgical therapy. This approach may help prevent chronic pain following spinal surgeries for different underlying pathologies.

These considerations are consistent with the findings of Wu et al., who reported that 29% of patients with chronic back pain after surgical treatment may experience problems due to factors such as insufficient or excessive decompression [11].

Our analysis also shows that ultimately only about 20% of the patients who were hospitalized for subacute and chronic lumbar spine complaints underwent spinal surgery at our clinic. This suggests that patient-specific injections led to long-lasting pain reduction in both MSP and SSP patients, potentially avoiding unnecessary lumbar spine surgeries.

However, this study has several limitations. The data were collected retrospectively, which limited our control over variables that could influence treatment outcomes and may have introduced bias. Our pain assessment method, which used a simple binary (yes/no) patient-reported outcome measure, lacks the nuance and detail that more comprehensive pain scales could offer. While this approach allowed for consistent data collection in a retrospective setting, it may have oversimplified patients’ pain experiences and missed subtle changes in pain levels. Future studies should consider employing more detailed pain assessment tools, such as the Numeric Rating Scale (NRS) or Visual Analog Scale (VAS), to capture a more comprehensive picture of pain reduction following interventions. Additionally, the effectiveness of the infiltrations was assessed based on patient-reported pain relief without quantifying the degree of pain reduction, which could also have led to bias. Furthermore, the number of patients in some subgroups, particularly those with specific spinal pathologies (SSP), may have been too small to draw definitive conclusions. As a single-center study, the results may have been influenced by local practices or specific patient demographics and may not be generalizable to other clinics. Finally, the correlation between imaging findings and clinical outcomes may have been incomplete, complicating the interpretation of the effectiveness of the injections.

## Conclusion

The targeted application of spinal infiltration is crucial in the treatment of complex degenerative lumbar spine diseases, offering a dual benefit: it helps accurately identify the origin of symptoms in cases with various pathologies and reduces the need for surgical interventions. This also applies to patients with a specific pathology in the lumbar spine. This approach not only improves patient outcomes by minimizing invasive procedures but also aligns with a patient-centered, conservative treatment paradigm for spinal disorders.

## Data Availability

The analyzed data are included in this manuscript. Original data can be requested from the corresponding author upon reasonable request.

## Abbreviations

BMI: Body mass index
EI: Epidural interlaminar
FJ: Facet joint
HIP: Hip joint
LP: Lumbar paraspinal
LSS: Lumbar spinal stenosis
MRI: Magnetic resonance imaging
MSP: Multiple spinal pathologies
SIJ: Sacroiliac joint
SSP: Specific spinal pathology
TEI: Transforaminal epidural injection
vs.: Versus
